# Perinatal outcomes after admission with COVID-19 in pregnancy: a UK national cohort study

**DOI:** 10.1038/s41467-024-47181-z

**Published:** 2024-04-15

**Authors:** Hilde Marie Engjom, Rema Ramakrishnan, Nicola Vousden, Kathryn Bunch, Edward Morris, Nigel Simpson, Chris Gale, Pat O’Brien, Maria Quigley, Peter Brocklehurst, Jennifer J. Kurinczuk, Marian Knight

**Affiliations:** 1 National Perinatal Epidemiology Unit, Nuffield Department of Population Health, University of Oxford, Oxford, OX3 7LF UK; 2 Division of Physical and Mental Health, Norwegian Institute of Public Health, 5015 Bergen, Norway; 3 Norfolk and Norwich University Hospital, Colney Lane, Norwich, NR4 7UY UK; 4 Department of Women’s and Children’s Health, School of Medicine, University of Leeds, Leeds, LS2 9JT UK; 5 Neonatal Medicine, School of Public Health, Faculty of Medicine, London, London, UK, SW7 2BX and Centre for Paediatrics and Child Health, Imperial College, London, SW7 2AZ UK; 6 Institute for Women’s Health, University College London, London, UK; 7 Birmingham Clinical Trials Unit, Institute of Applied Health Research, University of Birmingham, Birmingham, UK

**Keywords:** Viral infection, Epidemiology, Paediatric research, SARS-CoV-2

## Abstract

There are few population-based studies of sufficient size and follow-up duration to have reliably assessed perinatal outcomes for pregnant women hospitalised with SARS-CoV-2 infection. The United Kingdom Obstetric Surveillance System (UKOSS) covers all 194 consultant-led UK maternity units and included all pregnant women admitted to hospital with an ongoing SARS-CoV-2 infection. Here we show that in this large national cohort comprising two years’ active surveillance over four SARS-CoV-2 variant periods and with near complete follow-up of pregnancy outcomes for 16,627 included women, severe perinatal outcomes were more common in women with moderate to severe COVID-19, during the delta dominant period and among unvaccinated women. We provide strong evidence to recommend continuous surveillance of pregnancy outcomes in future pandemics and to continue to recommend SARS-CoV-2 vaccination in pregnancy to protect both mothers and babies.

## Abstract (web summary)

The impacts of maternal SARS-CoV-2 infection on newborns are not well understood. Here, the authors perform a national cohort study using data on births in the UK from March 2020—March 2022 and find that moderate to severe maternal COVID-19 was associated with adverse perinatal outcomes.

## Introduction

During the course of the pandemic, evolving variants of SARS-CoV-2 were associated with higher risk of severe maternal disease and adverse perinatal outcomes, but short observation time and lack of outcome data for continuing pregnancies were likely to bias results towards pregnancies ending at an earlier gestation and with more severe perinatal outcomes^1–4^.

The latest update of the World Health Organization (WHO) systematic review of coronavirus disease in pregnancy included studies published up to and including the alpha-dominant period^5^. Few of the studies in the WHO review were population-based or based on European data, and concerns have been raised about the conduct of other systematic reviews and the quality of included studies^6^.

Several of the larger multinational registry studies of COVID-19 in pregnancy lacked information about the source population and relied on passive, voluntary reporting^7–9^, and studies linking national routine health registry data have been unclear whether hospital admission was related to COVID-19 or pregnancy complications^10–13^. Robust evidence is needed to inform the care of pregnant women and counselling regarding vaccination^14–16^, particularly given the current increase in hospitalisations due to the omicron (XBB) lineage viruses and new emerging variants such as “pirola“ (BA.2.86)^17,18^.

Therefore, this study aimed to assess perinatal outcomes for mothers admitted to hospital with SARS-CoV-2 infection using a population-based national cohort with comprehensive follow-up.

## Results

**Fig. 1:**
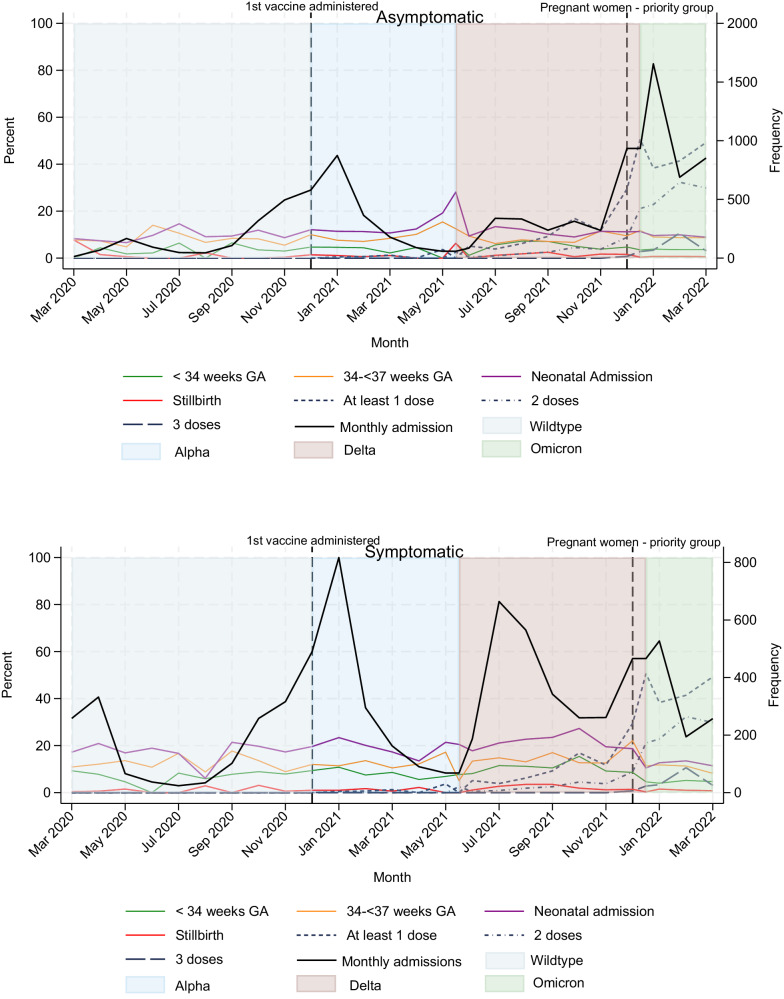
Monthly cumulative incidence (%) of perinatal outcomes. Stillbirth, preterm birth (<34 weeks’ and 34^+0^ –36^+6^  weeks) and admission to a neonatal unit is shown by symptom status with asymptomatic women in the upper panel and symptomatic women in the lower panel. Number of women admitted to hospital for each month from March 2020 to March 2022 is shown on *Y* axis to the right. Vaccination status among included women by minimum number of doses recorded; at least 1 dose, 2 doses or 3 doses (%) is shown in the dotted lines. The vertical dashed lines indicate key vaccination policies: The first COVID-19 vaccine in pregnancy was administered in the U.K. in December 31, 2020, and pregnant women were included as priority group by the Joint Committee on Vaccination and Immunisation (JCVI) in the U.K in December 2021. The dominant SARS-CoV-2 variant is indicated by the background colour; wild-type, alpha, delta and omicron.

Among a source population of ~1.5 million maternities^19–21^, 16,627 women were included (Supplementary Fig. S1). Their sociodemographic characteristics and vaccination status are shown by symptom status and severity in Table 1. A higher proportion of women with moderate to severe COVID-19 were overweight or obese, were of a minority ethnic background, or were unvaccinated compared with women with asymptomatic or mild infection (Table 1). Medical conditions, pregnancy characteristics as well as COVID-19 directed treatment is shown in Table 2. Women with moderate to severe infection were more likely to have asthma or diabetes and be admitted prior to 37 weeks’ gestation (77.3%) compared with asymptomatic women (24.9%) and women with mild infection (51.3%) (Table 2). Vaccination coverage increased gradually amongst women admitted with SARS-CoV-2 from May 2021 onwards (Fig. 1).

**Table 1:** Sociodemographic characteristics and vaccination status of pregnant women admitted with SARS-CoV-2, by symptom group, March 1, 2020, to March 31, 2022, United Kingdom

Symptom group	Asymptomatic (*N* = 9374)	Mild infection (*N* = 4901)	Moderate to severe infection^a^ (*N* = 2352)
Age (years)—no. (%)			
<20	231 (2.5)	100 (2.0)	21 (0.9)
20-34	6685 (71.5)	3583 (73.2)	1544 (65.8)
≥35	2438 (26.1)	1210 (24.7)	782 (33.3)
Missing	20	8	5
Body Mass Index (BMI) (kg/m^2^) - no. (%)			
Underweight (<18.5)	229 (2.5)	101 (2.1)	17 (0.8)
Normal (18.5 to <25)	3599 (39.7)	1829 (38.5)	529 (23.4)
Overweight (25 to <30)	2702 (29.8)	1467 (30.9)	714 (31.6)
Obese ( ≥ 30)	2528 (27.9)	1357 (28.5)	1000 (44.3)
Missing	316	147	92
Woman or partner in paid work - no. (%)	7010 (74.8)	3788 (77.3)	1791 (76.2)
Ethnic Group - no. (%)			
White	6107 (66.7)	3134 (65.4)	1350 (58.9)
Asian	1689 (18.5)	934 (19.5)	519 (22.6)
Black	825 (9.0)	418 (8.7)	254 (11.1)
Chinese/Other	282 (3.1)	178 (3.7)	110 (4.8)
Mixed	249 (2.7)	129 (2.7)	59 (2.6)
Missing	222	108	60
Current smoking - no. (%)	1547 (17.0)	626 (13.1)	178 (7.8)
Missing	294	138	68
Vaccination status - no. (%)			
Unvaccinated	6099 (65.1)	3424 (69.9)	1785 (75.9)
1 dose	620 (6.6)	271 (5.5)	72 (3.1)
2 doses	835 (8.9)	290 (5.9)	41 (1.7)
3 doses	140 (1.5)	53 (1.1)	4 (0.2)
Not known/ not documented	1680 (17.9)	863 (17.6)	450 (19.1)

^a^Moderate to severe COVID-19 was defined as one or more of the following: maternal death, intensive care unit admission, peripheral oxygen saturation below 95% at admission, pneumonia on imaging, or need for respiratory support with either oxygen, high flow nasal cannula or continuous positive pressure mask, mechanical ventilation or extracorporeal membrane oxygenation.

^b^27 (0.3%) asymptomatic women needed respiratory support or were admitted to ICU for other reasons and one of them died from other causes than COVID-19.

**Table 2:** Maternal medical conditions, pregnancy characteristics and COVID-19 severity of pregnant women admitted with SARS-CoV-2, by symptom group, March 1, 2020, to March 31, 2022, United Kingdom

Symptom group	Asymptomatic (*N* = 9374)	Mild infection (*N* = 4901)	Moderate to severe infection^a^ (*N* = 2352)
Pre-existing medical conditions - no. (%)			
Asthma	552 (5.9)	375 (7.7)	231 (9.8)
Hypertension	155 (1.7)	80 (1.6)	55 (2.3)
Cardiac disease	114 (1.2)	68 (1.4)	38 (1.6)
Diabetes	132 (1.4)	71 (1.5)	60 (2.6)
Medical conditions during pregnancy - no. (%)			
Pre-eclampsia	141 (1.5)	73 (1.5)	49 (2.1)
Gestational diabetes	595 (6.4)	354 (7.2)	263 (11.2)
Multiparous - no. (%)	5749 (61.9)	3038 (62.4)	1626 (69.8)
Missing	90	35	22
Multiple pregnancy - no. (%)	140 (1.5)	96 (2.0)	51 (2.2)
Gestation at admission (weeks^days^) - no. (%)			
<22	463 (5.0)	468 (9.6)	194 (8.3)
22-27^+6^	300 (3.2)	445 (9.1)	415 (17.8)
28-33^+6^	639 (6.9)	863 (17.7)	738 (31.7)
34−36^+6^	915 (9.8)	729 (14.9)	455 (19.5)
37 or more	6987 (75.1)	2374 (48.7)	529 (22.7)
Missing	70	22	21
Evidence of pneumonia on imaging - no. (%)	-	-	1695 (72.1)
Respiratory support required - no. (%)	-^b^	-	1709 (76.2)
Intensive Care Unit admission - no. (%)	-^b^	-	771 (32.8)
Maternal Death - no. (%)	1 (0.01)	-	38 (1.6)

^a^Moderate to severe COVID-19 was defined as one or more of the following: maternal death, intensive care unit admission, peripheral oxygen saturation below 95% at admission, pneumonia on imaging, or need for respiratory support with either oxygen, high flow nasal cannula or continuous positive pressure mask, mechanical ventilation or extracorporeal membrane oxygenation.

^b^ 27 (0.3%) asymptomatic women needed respiratory support or were admitted to ICU for other reasons and one of them died from other causes than COVID-19.

Of the 16,386 (98.6%) women with known pregnancy outcomes, 1.9% (*n* = 318) experienced a pregnancy loss (Supplementary Fig. S1) and the remaining 16,068 women gave birth to 16,351 infants of whom 190 were stillborn. Pregnancy outcomes for symptomatic women are shown by variant and severity in Table 3, and for asymptomatic women in Supplementary Table S1. Women with moderate to severe COVID-19 were more likely to give birth prior to 37 weeks, have an expedited birth due to COVID-19 and a pre-labour caesarean birth than women with mild disease.

**Table 3:** Maternal and pregnancy outcomes for women with symptomatic SARS-CoV-2 by severity, March 1, 2020, to March 31, 2022, United Kingdom

Severity	Mild infection (*N* = 4901)	Moderate to severe infection^a^ (*N* = 2352)
Pregnancy outcome – no. (%)		
Birth	4710 (96.1)	2264 (96.3)
Pregnancy loss	92 (1.9)	26 (1.1)
Birth outcome unknown	99 (2.0)	62 (2.6)
Gestation at birth (weeks^+days^)^b^ - no. (%)		
22^+0^ – 27^+6^	47 (1.0)	49 (2.2)
28^+0^ – 33^+6^	180 (3.8)	305 (13.6)
34^+0^ – 36^+6^	441 (9.4)	395 (17.6)
37^+0^ or more	4022 (85.8)	1490 (66.6)
Missing	20	24
Birth expedited due to COVID-19^b^ - no. (%)	80 (1.7)	601 (26.6)
Mode of birth^b^ - no. (%)		
Pre-labour Caesarean	1246 (26.6)	1129 (50.4)
Caesarean after labour onset	702 (15.0)	260 (11.6)
Operative vaginal	491 (10.5)	141 (6.3)
Spontaneous vaginal	2239 (47.9)	712 (31.8)
Missing	32	21

^a^Moderate to severe COVID-19 defined as one or more of the following. Maternal death, intensive care unit admission, peripheral oxygen saturation below 95% at admission, pneumonia on imaging, or need for respiratory support with oxygen, high flow nasal cannula or continuous positive pressure mask, mechanical ventilation or extracorporeal membrane oxygenation.

^b^Pregnancy loss excluded from denominator.

The perinatal outcomes for births to symptomatic women are shown in tables four to six and for births to asymptomatic women in Supplementary Table S2. Amongst the 7116 infants born to 6974 symptomatic women, 1.6% (*n* = 111) were stillborn (Table 4) and 8.7% (*n* = 617) were born prior to 34 weeks’ gestation (Table 5).

**Table 4:** Stillbirths to women admitted to hospital with symptomatic SARS-CoV-2 by dominant variant period and severity of maternal infection, March 1, 2020, to March 31, 2022, United Kingdom

SARS-CoV-2 dominant variant	Wild-type period		Alpha period		Delta period		Omicron period	
Severity^a^	Mild (*N* = 1067)	Moderate to severe^a^ (N = 370)	Mild (*N* = 1220)	Moderate to severe^a^ (*N* = 678)	Mild (*N* = 1420)	Moderate to severe^a^ (*N* = 1055)	Mild (*N* = 1098)	Moderate to severe^a^ (*N* = 208)
Stillbirths - no. (%)	9 (0.8)	6 (1.6)	12 (1.0)	9 (1.3)	42 (3.0)	20 (1.9)	8 (0.7)	5 (2.4)
Model 1^b^: RR (95% CI)	[Ref]	1.93 (0.68–5.38)	1.17 (0.49–2.76)	1.57 (0.63–3.95)	3.49 (1.70–7.18)	2.25 (1.03–4.92)	0.86 (0.32–2.34)	2.86 (0.96–8.46)
Model 2^c^: RR (95% CI)	[Ref]	1.90 (0.61–5.88)	1.20 (0.49–2.97)	1.68 (0.63–4.51)	3.57 (1.66–7.67)	2.41 (1.03–5.60)	0.96 (0.35–2.66)	2.62 (0.79–8.69)
Model 3^d^: RR (95% CI)	NA	NA	[Ref]	0.99 (0.33–3.01)	2.82 (1.29–6.16)	1.89 (0.81–4.40)	0.79 (0.25–2.47)	2.07 (0.64–6.76)

Abbreviations: *GA* gestational age, *RR* risk ratio, *CI* confidence interval, *NA* Not available.

^a^Moderate to severe COVID-19 defined as one or more of the following. maternal death, intensive care unit admission, peripheral oxygen saturation below 95% at admission, pneumonia on imaging, or need for respiratory support with oxygen, high flow nasal cannula or continuous positive pressure mask, mechanical ventilation or extracorporeal membrane oxygenation.

^b^Model 1: Variant + severity + interaction term for variant and severity.

^c^Model 2: Model 1  + maternal age, ethnicity, employment status, body mass index, multiple pregnancy, smoking, parity, pre-existing medical conditions, pre-eclampsia, gestational diabetes, and gestational age at admission.

^d^Model 3: Model 2 + vaccination status. Vaccination started from January 1, 2021, therefore mild alpha infection was used as the reference group.

**Table 5:** Preterm births to women admitted to hospital with symptomatic SARS-CoV-2 by dominant variant period and severity of maternal infection, March 1, 2020, to March 31, 2022, United Kingdom

SARS-CoV-2 dominant variant	Wild-type period		Alpha period		Delta period		Omicron period	
Severity^a^	Mild (*N* = 1067)	Moderate to severe^a^ (*N* = 370)	Mild (*N* = 1220)	Moderate to severe^a^ (*N* = 678)	Mild (*N* = 1420)	Moderate to severe^a^ (*N* = 1055)	Mild (*N* = 1098)	Moderate to severe^a^ (*N* = 208)
Preterm live birth^e^ - no. (%)								
<34 weeks’ GA	56 (5.3)	57 (15.5)	56 (4.6)	121 (18.1)	92 (6.5)	177 (16.9)	41 (3.7)	17 (8.2)
Model 1^b^: RR (95% CI)	[Ref]	3.74 (2.52–5.55)	0.87 (0.59–1.27)	4.41 (3.15–6.17)	1.27 (0.90–1.79)	4.26 (3.10–5.85)	0.69 (0.46–1.04)	1.83 (1.04–3.24)
Model 2^c^: RR (95% CI)	[Ref]	3.46 (2.24–5.34)	0.84 (0.56–1.27)	5.03 (3.51–7.20)	1.37 (0.95–1.97)	4.94 (3.52–6.94)	0.66 (0.42–1.03)	2.02 (1.13–3.63)
Model 3^d^: RR (95% CI)	NA	NA	[Ref]	6.84 (4.42–10.59)	1.92 (1.24–2.96)	7.01 (4.65–10.59)	0.98 (0.59- 1.63)	2.98 (1.57–5.65)
34^+0^ – 36^+6^ weeks’ GA	104 (9.8)	65 (17.7)	120 (9.9)	105 (15.7)	153 (10.8)	201 (19.2)	101 (18.0)	41 (19.8)
Model 1^b^: RR (95% CI)	[Ref]	2.30 (1.63–3.23)	1.00 (0.76–1.32)	2.06 (1.54–2.77)	1.14 (0.87–1.48)	2.60 (2.01–3.37)	0.92 (0.69–1.22)	2.38 (1.59–3.56)
Model 2^c^: RR (95% CI)	[Ref]	2.36 (1.62–3.42)	1.00 (0.74–1.34)	2.38 (1.73–3.26)	1.18 (0.89–1.57)	2.92 (2.22–3.86)	1.04 (0.77–1.42)	2.55 (1.67–3.87)
Model 3^d^: RR (95% CI)	NA	NA	[Ref]	2.32 (1.63–3.31)	1.23 (0.90–1.67)	3.00 (2.21–4.06)	1.20 (0.85–1.69)	2.81 (1.81–4.35)

Abbreviations: *GA* gestational age, *RR* risk ratio, *CI* confidence interval, *NA* Not available.

^a^Moderate to severe COVID-19 defined as one or more of the following. maternal death, intensive care unit admission, peripheral oxygen saturation below 95% at admission, pneumonia on imaging, or need for respiratory support with oxygen, high flow nasal cannula or continuous positive pressure mask, mechanical ventilation or extracorporeal membrane oxygenation.

^b^Model 1: Variant + severity + interaction term for variant and severity.

^c^Model 2: Model 1 + maternal age, ethnicity, employment status, body mass index, multiple pregnancy, smoking, parity, pre-existing medical conditions, and pre-eclampsia.

^d^Model 3: Model 2 + vaccination status. Vaccination started from January 1, 2021, so mild alpha infection was used as reference group.

^e^45 infants born to symptomatic women had missing data for gestational age at birth.

The absolute risk of stillbirth among symptomatic women was 0.8% for mild infection in the wild-type period and 1.6%, 1.3%, 1.9% and 2.4% with moderate to severe maternal infection in the wild-type, alpha, delta and omicron variant periods, respectively (Table 4). Compared to women with mild infection during the wild-type period, there was no evidence for a statistically significant increased risk of stillbirth for women with moderate to severe infection, except for during the delta period, noting that smaller numbers impacted the study power to detect differences as statistically significant (Table 4). After adjusting for maternal risk factors, there was an increased risk of stillbirth during the delta period irrespective of infection severity (mild RR 3.57; 95%CI 1.66 to 7.67 and moderate to severe RR 2.41; 95%CI 1.03 to 5.60) when compared with mild infection during the wild-type period. (Table 4, model 2).

The risk of being born prior to 34 weeks’ gestation was 7.9% in the wild-type period and 9.4% with alpha (crude RR 1.21; 95%CI 0.94 to 1.55), 10.9% with delta (crude RR 1.49; 95%CI 1.18 to 1.87) and 4.5% with omicron (crude RR 0.53; 95%CI 0.38 to 0.74) (Supplementary Table S3). The risk of being born at 34^+0^-36^+6^ weeks’ gestation was 11.8% in the wild-type period and 11.9% with alpha (crude RR 1.03; 95%CI 0.83 to 1.87), 14.4% with delta (crude RR 1.31; 95%CI 1.07 to 1.59) and 10.9% with omicron (crude RR 0.87; 95%CI 0.69 to 1.11) (Supplementary Table S3). Compared with births among women with mild infection, infants born to women with moderate to severe infection had a fourfold increased risk of birth before 34 weeks’ gestation and a nearly doubled risk of birth at 34^+0^-36^+6^  weeks’ gestation in the crude risk ratio analyses (Supplementary Table S4).

When the association was examined by variant period and infection severity, the risk of being born preterm prior to 34 weeks’ gestation was 5.3% with mild maternal infection during the wild-type period, and 15.5%, 18.1%, 16.9% and 8.2% with moderate to severe maternal infection in the wild-type, alpha, delta and omicron variant periods, respectively (Table 5). We found evidence of a multiplicative interaction between variant period and infection severity on the risk of preterm birth. The risk of birth before 34 weeks’ was five times higher among infants born to women with moderate to severe infection during the alpha and delta periods, compared with women with mild infection during the wild-type period, after adjusting for maternal risk factors (alpha RR 5.03; 95%CI 3.51 to 7.20; delta RR 4.94; 95%CI 3.52 to 6.94) (Table 5, model 2) whereas this risk was doubled during the omicron period (RR 2.02; 95%CI 1.13 to 3.63) (Table 5, model 2). The risk increase was two-to-threefold higher for birth between 34^+0^ to 36^+6^  weeks’ gestation among women with moderate to severe infection across all periods when compared to mild wild-type infection (Table 5, model 2).

We also found evidence of a multiplicative interaction between variant period and infection severity on neonatal unit admission. Compared with women with mild infection in the wild-type period, analyses accounting for both severity and variant in a model adjusted for maternal risk factors and gestational age at birth (model 2) showed 1.5 times higher risk of neonatal unit admission across all variant periods in neonates born to women with moderate to severe infection (Table 6, model 2). These patterns for preterm birth and neonatal unit admission persisted after accounting for vaccination status (Table 5 and Table 6, model 3). Neonatal deaths at <7 days of age were rare, with a total of 18 deaths among babies born to symptomatic women (Table 6).

**Table 6:** Neonatal outcomes for babies of women admitted to hospital with symptomatic SARS-CoV-2 by dominant variant period and severity of maternal infection, March 1, 2020, to March 31, 2022, United Kingdom

SARS-CoV-2 dominant variant	Wild-type period		Alpha period		Delta period		Omicron period	
Severity^a^	Mild (*N* = 1067)	Moderate to severe^a^ (*N* = 370)	Mild (*N* = 1220)	Moderate to severe^a^ (*N* = 678)	Mild (*N* = 1420)	Moderate to severe^a^ (*N* = 1055)	Mild (*N* = 1098)	Moderate to severe^a^ (*N* = 208)
Neonatal unit admission^e^ - no. (%)	144 (13.6)	120 (33.1)	160 (13.3)	231 (34.5)	180 (13.1)	349 (33.7)	105 (9.6)	50 (24.5)
Model 1^b^: RR (95% CI)	[Ref]	2.43 (1.97–3.00)	0.98 (0.79- 1.20)	2.53 (2.11–3.05)	0.96 (0.78–1.18)	2.47 (2.08–2.94)	0.71 (0.56–0.90)	1.80 (1.35–2.39)
Model 2^c^: RR (95% CI)	[Ref]	1.61 (1.32–1.96)	0.99 (0.81- 1.21)	1.61 (1.35–1.93)	0.88 (0.73–1.08)	1.52 (1.28–1.81)	0.76 (0.61–0.95)	1.51 (1.16–1.97)
Model 3^d^: RR (95% CI)	NA	NA	[Ref]	1.60 (1.30–1.97)	0.92 (0.73–1.14)	1.57 (1.28–1.94)	0.77 (0.60–0.99)	1.55 (1.16–2.07)
Neonatal death^f^ - no. (%)^g^	4 (0.4)	1 (0.3)	0	2 (0.3)	6 (0.4)	3 (0.3)	2 (0.2)	0

Abbreviations: *GA* gestational age, *RR* risk ratio, *CI* confidence interval, *NA* Not available.

^a^Moderate to severe COVID-19 defined as one or more of the following. maternal death, intensive care unit admission, peripheral oxygen saturation below 95% at admission, pneumonia on imaging, or need for respiratory support with oxygen, high flow nasal cannula or continuous positive pressure mask, mechanical ventilation or extracorporeal membrane oxygenation.

^b^Model 1: Variant + severity + interaction term for variant and severity.

^c^Model 2: Model 1 + gestational age at birth, parity, and pre-existing medical conditions.

^d^Model 3: Model 2 + vaccination status. Vaccination started from January 1, 2021, so mild alpha infection was used as reference group.

^e^112 infants born to symptomatic women had missing data for admission to neonatal unit.

^f^75 infants born to symptomatic women had missing data for neonatal death.

^g^Risk ratios were not calculated due to low numbers.

Amongst 5185 births to symptomatic women from January 1, 2021 onwards, when vaccination was recommended for pregnant women in risk groups, there were 91 stillbirths among symptomatic women; 91.2% (83/91) occurred to women with no documented vaccine or unknown vaccination status (Table 7). Women who were unvaccinated or had unknown vaccination status also gave birth to 92.1% (422/458) of the infants born before <34 weeks’ gestation in the symptomatic group. Perinatal outcomes and maternal vaccination status for asymptomatic women are reported in online supplementary Table S5; 75% (49/65) of the stillbirths and 78.1% (249/319) of infants born prior to 34 weeks were born to women with no documented vaccination or unknown vaccination status.

**Table 7:** Perinatal outcomes in births to women with symptomatic SARS-CoV-2 admitted to hospital by number of documented maternal vaccination doses, from January 1, 2021, to March 31, 2022, United Kingdom

Vaccination status	Unvaccinated (*N* = 3184)	Vaccine status unknown (*N* = 1275)	1 dose (*N* = 347)	2 doses (*N* = 319)	3 doses (*N* = 60)
Stillbirth - no. (%)	64 (2.0)	19 (1.5)	3 (0.9)	5 (0.6)	0
Preterm births^a^ - no. (%)					
<34 weeks	299 (9.5)	123 (9.7)	13 (3.8)	20 (6.3)	3 (5.0)
34^+0^ –36^+6^ weeks’	443 (14.0)	152 (12.0)	34 (9.9)	26 (8.2)	7 (11.7)
Admission to Neonatal Unit^b^- no. (%)	620 (19.9)	270 (21.5)	40 (11.7)	40 (12.7)	9 (15.0)
Neonatal Death^c^ - no. (%)	6 (0.2)	3 (0.2)	2 (0.6)	1 (0.3)	0

^a^45 infants born to symptomatic women had missing data for gestational age at birth.

^b^112 infants born to symptomatic women had missing data for admission to neonatal unit.

^c^77 infants born to symptomatic women had missing data for neonatal death.

## Discussion

This national cohort study of pregnant women admitted to hospital with SARS-CoV-2 shows that compared with women with mild infection in the wild-type period there was increased risk of stillbirth for women with mild and moderate to severe infection during the delta period. The risk of preterm birth was increased for babies born to women with moderate to severe infection across all periods. Omicron was considered to be a variant with lower risk of adverse perinatal outcomes^22^, yet we observed a threefold increase in risk of birth prior to 34  weeks’ gestation for women with moderate to severe COVID-19 in this period compared with mild infection in the wild-type period, after accounting for vaccination status. Unvaccinated women appeared to more frequently experience stillbirth and preterm birth.

The key strengths of this study are that birth outcomes were available for almost all the included women. The large sample size provides sufficient statistical power to detect a difference in stillbirth during the delta period. We observed an increase in risk of preterm birth with moderate to severe maternal infection across all variant periods, demonstrating the merit of a nuanced analysis taking variant and severity into account.

In addition, the availability of data about symptom status, severity of infection and vaccination status allowed robust regression models. The UKOSS system of active and uniform case identification across all pregnant women admitted to hospital in the UK that tested positive for SARS-CoV-2 reduces the risk of selection bias compared with other studies. Finally, the clinical information reduced the risk of misclassification bias, such as attributing adverse outcomes associated with pregnancy-related admission incorrectly to COVID-19.

Several limitations also need to be considered. First, perinatal outcomes in births to pregnant women with SARS-CoV-2 who were not admitted to hospital at the time of infection could not be assessed. There would likely be few adverse outcomes in this group, as studies from other countries did not find an increased risk of adverse perinatal outcomes with a test-delivery interval exceeding 10 days^23^. Second, we have included time periods when the variants of SARS-CoV-2 were dominant as proxy for individual variant sequencing data which were not available. Third, whilst providing robust data about the prevalence of stillbirth in women with COVID-19, we do not have clinical information to assess the cause of death and differentiate iatrogenic and spontaneous nature of the preterm birth.

Despite the inclusion of many associated confounders, residual confounding from other factors, including health beliefs and health seeking behaviours, and time-dependent changes that could contribute to increased risk of stillbirth and preterm birth remain. For example, indirect impacts of the pandemic such as competing hospital pressures, differing practice around intrapartum fetal monitoring for women with COVID-19, or delay in seeking care may contribute to the higher proportion of stillbirths observed in women with COVID-19 during the delta period, but this does not explain the increase in preterm births.

The proportion of stillbirths observed in women with asymptomatic infection during the wild-type period was comparable to nationally-reported figures in the UK (4/1000 total births versus 3.5/1000 total births, respectively), whereas rates in women with mild symptomatic infection in the wildtype period were 8/1000 total births^24^. Whilst a strength of this study is the comparison of variants over time, it is limitation of this study that rates in symptomatic women could not be compared to women without COVID-19 infection due to the absence of a control data. Recent UK-wide surveillance of perinatal deaths identified an increase in the perinatal mortality rate during 2021^24^. The current study indicates that COVID-19 in unvaccinated pregnant women could have contributed to this increase.

This study significantly adds to the field since previous cohort studies that ended during earlier variant periods reported stillbirth rates comparable with pre-pandemic periods^1,12,25^, thus demonstrating the need for ongoing surveillance of perinatal outcomes during a pandemic to inform care. Similarly, other studies with shorter^26^ or unclear^27^ duration of follow up after admission with infection have had insufficient data to reliably compare severe perinatal outcomes and did not account for severity of maternal infection.

By January 2022, 50.6% of the women who gave birth in England had received two vaccine doses^28^. Previous cohort analyses have demonstrated the benefit of maternal SARS-CoV-2 vaccination to prevent hospital admission due to COVID-19 in pregnant women^29^, reduce moderate to severe maternal COVID-19^2,4^, and reduce infant hospitalization for COVID-19, severe neonatal morbidity and death^3,30,31^. The current study clearly demonstrates the benefit of maternal vaccination on perinatal outcomes, showing a large proportion of stillbirths in women with no documented vaccination in both those with and without any symptoms of COVID-19.

Preterm birth is also associated with adverse short-term^32^ and long-term^33,34^ health outcomes and the need for neonatal unit admissions increases demand and economic impact on the health services^35^. With ongoing increasing SARS-CoV-2 infections, it is paramount to ensure that pregnant women are prioritised for vaccination, and that messaging around safety and access to vaccines is clear and readily available.

In conclusion, this large national cohort study comprising 2 years’ active surveillance over four SARS-CoV-2 variant periods and with near complete follow-up of pregnancy outcomes for included women, shows that severe perinatal outcomes were more common in women with moderate to severe COVID-19, during the delta dominant period and among unvaccinated women. We provide strong evidence to recommend continuous surveillance of pregnancy outcomes in future pandemics and to continue to recommend vaccination in pregnancy to protect both mothers and babies.

## Methods

### Study design and oversight

The United Kingdom Obstetric Surveillance System (UKOSS) has a source population of all women giving birth or being admitted for obstetric specialist care to one of the 194 consultant-led maternity units in England, Northern Ireland, Scotland and Wales, ~720,000 maternities annually^1,19–21^. A protocol for active surveillance of pregnant women admitted to hospital with viral infection was planned in 2012, approved by the ethical review board (HRA NRES Committee East Midlands), Nottingham 1 (Ref. Number: 12/EM/0365) and hibernated (10.1186/ISRCTN40092247). The protocol was activated for Severe Acute Respiratory Syndrome Coronavirus 2 (SARS-CoV-2) from March 1, 2020, and concluded on March 31, 2022. Routines were in place to ensure complete reporting (online supplementary)^3^, and follow-up information about birth outcomes up to December 31, 2022, were retrieved from clinical records until April 24, 2023. Maternal and perinatal deaths were cross-checked with the MBRRACE-UK mortality surveillance. (https://www.npeu.ox.ac.uk/mbrrace-uk). The corresponding author vouches for the accuracy and completeness of the data and reporting. Patients and public were part of the UKOSS steering committee and involved in study design, oversight, reporting and dissemination of study findings but not in the conduct of the study. The funder played no role in study design; in the collection, analysis, and interpretation of data; in the writing of the report; nor in the decision to submit the paper for publication.

### Study population and groups

Pregnant women were included if admitted to hospital with a positive SARS-CoV-2 reverse transcriptase polymerase chain reaction (PCR) test within 7 days of admission, during admission or up to 2 days after giving birth. Women were further classified according to their SARS-CoV-2 symptoms (symptomatic/asymptomatic); severity of infection (mild/moderate to severe); and the dominant viral variant in UK at the time of the PCR test (wild-type March 1 to November 30, 2020; alpha December 1, 2020, to May 15 2021; delta May 16 to December 14, 2021; omicron (BA.1 and BA.2 variant) December 15, 2021, to March 31 2022)^36^.

### Severity of maternal Covid-19

Moderate to severe maternal COVID-19 was defined according to modified WHO criteria as maternal death, maternal intensive care admission, peripheral oxygen saturation below 95% at admission, pneumonia on radiological imaging or need for respiratory support (either oxygen supplementation, non-invasive ventilation (high flow nasal oxygen or continuous positive airway pressure), mechanical ventilation or extracorporeal membrane oxygenation (ECMO))^37^.

### Maternal characteristics and medical risk factors

The sociodemographic characteristics and medical risk factors recorded were maternal age, maternal body mass index (kg/m^2^), employment, ethnic background, smoking, pre-existing medical conditions (no medical conditions (reference) vs asthma, hypertension, cardiac disease or diabetes), preeclampsia, gestational diabetes, parity, plurality and gestational age at admission (categorised by completed weeks into <22, 22 to 27, 28-33, 34−36 and ≥37 weeks). Vaccination status was recorded from January 2021 after vaccination was recommended for pregnant women in risk groups in the UK from December 22, 2020^38–40^.

### Pregnancy and perinatal outcomes

Pregnancy outcomes examined were pregnancy loss ( < 24 weeks’ gestation)^41^, gestational age at birth, expedited birth due to COVID-19, and mode of birth (spontaneous vaginal, operative vaginal, caesarean section prior to or during labour). The perinatal outcomes were stillbirth at ≥ 24 weeks’ gestation^41^, preterm birth (<34 weeks’ and 34^+0^ to 36^+6^ weeks’ gestation), neonatal unit admission, and neonatal death within 7 days after birth.

### Statistical analyses

Percentages and frequencies were computed by symptom group, and for symptomatic women by severity of infection, dominant variant, and vaccination status. Where data were missing, percentages are presented as the proportion of cases known.

Risk ratios (RR) with 95% confidence intervals (CI) for stillbirth, preterm birth and admission to neonatal unit for births to symptomatic women were computed using symptomatic mild infection during the wild-type period as the reference category with the lowest absolute risk. Since severity and variant were known risk factors, we included a preplanned interaction analysis to assess the combined effect of these factors^2,4^. Several models were run: models with dominant variant only (crude RR), disease severity only (crude RR), and severity and variant along with an interaction term for both (interaction without covariates (model 1)); adjusted for selected covariates without vaccination status (model 2); and adjusted for selected covariates and vaccination status using mild infection during the alpha period as reference (model 3). The covariates were selected based on availability of information and evidence from the literature^3,42,43^.

Multilevel Poisson or multinomial regression model as appropriate was used with random intercept to account for clustering effect among multiple births. The multivariable model for stillbirth was adjusted for maternal age, ethnicity, employment status, body mass index, plurality, smoking, parity, medical conditions prior to or during pregnancy and gestational age at admission. The preterm birth model included the above except gestational age at admission, and the model for neonatal unit admission was adjusted for gestational age at birth, parity, and medical conditions. Statistical analyses were performed using STATA version 18 (Statacorp, TX, USA).

Study registration number: (10.1186/ISRCTN40092247)

Ethics approval: HRA NRES Committee East Midlands—Nottingham 1 (Ref. Number: 12/EM/0365). Information about ethnicity was self-determined according to the UK standard census categories (List of ethnic groups)—GOV.UK (ethnicity-facts-figures.service.gov.uk).

Rema Ramakrishnan, Hilde M Engjom, Nicola Vousden and Marian Knight analysed the data.

The study protocol was published at the study start and is available on the UKOSS website https://www.npeu.ox.ac.uk/ukoss/completed-surveillance/covid-19-in-pregnancy.

### Reporting summary

Further information on research design is available in the Nature Portfolio Reporting Summary linked to this article.

### Supplementary information

Supplementary Information

Peer Review File

Reporting Summary

## Data Availability

Data cannot be shared because of confidentiality issues and potential identifiability of sensitive data as identified in the Research Ethics Committee approval. Requests to access the data can be made by contacting the National Perinatal Epidemiology Unit data access committee via general@npeu.ox.ac.uk. The estimated response time for requests is 4 weeks. Data sharing outside the UK or the European Union may require consultation with the UK Health Research Authority.
