# Supplementary material for: Perinatal outcomes after admission with COVID-19 in pregnancy: a UK national cohort study
Source: Nat Commun. 2024 Apr 15;15:3234. doi: 10.1038/s41467-024-47181-z (PMC11018846; doi:10.1038/s41467-024-47181-z)
Supplement: Supplementary file 1 — Supplementary Information [file 41467_2024_47181_MOESM1_ESM.pdf]

Supplementary material

Table of content

The UK Obstetric Surveillance System -UKOSS 2

Supplementary Figure S1. Study population flow chart 3

Supplementary Table S1. Pregnancy outcomes for asymptomatic women. 4

Supplementary Table S2. Perinatal outcomes in births to asymptomatic women by dominant variant. 5

Supplementary Table S3. Crude risk ratios for perinatal outcomes in births to symptomatic women by dominant variant. 6

Supplementary Table S4. Crude risk ratios for perinatal outcomes in births to symptomatic women by severity of maternal infection. 8

Supplementary Table S5. Perinatal outcomes in births to asymptomatic women by maternal vaccine status. 9

References 10

The UK Obstetric Surveillance System -UKOSS

UKOSS is a research platform that was established in 2005. All 194 hospitals in the UK with a consultant led maternity unit collect population-based information about specific severe pregnancy complications.

Nominated reporting clinicians, facilitated by research midwives and nurses from the UK's National Institute of Health Research Clinical Research Network, sent notification of all pregnant women admitted to their hospital with confirmed SARS-CoV-2 infection to UKOSS. In addition to receipt of real time notifications, hospitals that did not notify any admissions were confirmed to have zero admissions. The UKOSS team sent reminders to reporters who had sent notifications but had not returned data. Hospital admission was defined as an overnight hospital stay, or longer, for any cause, or admission of any duration to give birth.[1,2] Women were regarded as having confirmed SARS-CoV-2 if they were admitted during pregnancy and they had a positive test in the seven days prior to admission or during their admission. Women who only had a positive test during admission which was more than two days after giving birth were excluded. Information on women who died, or who had stillbirths or neonatal deaths, was cross checked with data from the organisation responsible for maternal and perinatal death surveillance in the UK (MBRRACE-UK: Mothers and Babies: Reducing Risk through Audits and Confidential Enquiries across the UK | MBRRACE-UK | NPEU (ox.ac.uk). Birth outcomes for women included up to March 31, 2022 but who were discharged while still pregnant, were retrieved from clinical records until April 24, 2023.

Supplementary Figure S1. Study population flow chart

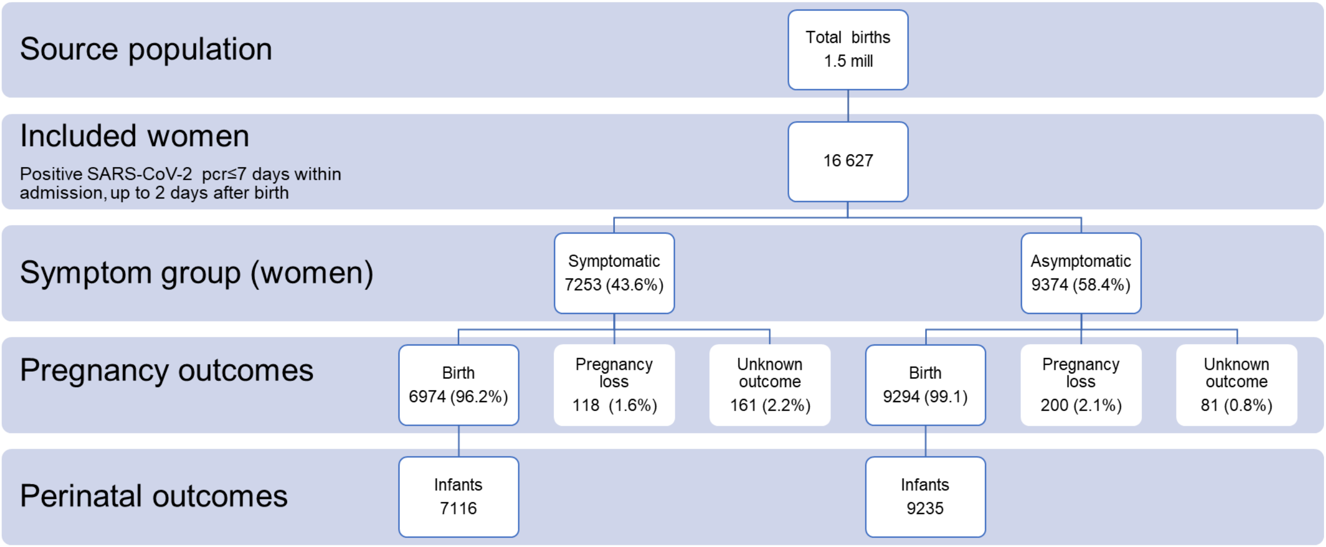

Supplementary Table S1. Pregnancy outcomes for asymptomatic women.

Table S1. Maternal and pregnancy outcomes for women with asymptomatic SARS-CoV-2 admitted to hospital from March 1, 2020, to March 31, 2022, United Kingdom.

Total pregnancies -no. (%)	9374
Births	9094 (97.0)
Pregnancy loss	200 (2.1)
Birth outcome unknown	80 (0.9)
Gestation at birth (weeks^{+days})* -no. (%)	
<22 weeks	1 (0.01)
22^{+0} – 27^{+6}	88 (1.0)
28^{+0} – 33^{+6}	268 (3.0)
34^{+0} – 36^{+6}	711 (7.9)
37^{+0} or more	7964 (88.2)
Missing	62
Mode of birth* -no. (%)	
Pre-labour Caesarean	2170 (24.0)
Caesarean after labour onset	1228 (13.6)
Operative vaginal	1006 (11.2)
Unassisted vaginal	4621 (51.2)
Missing	69

* Excluding pregnancy loss from denominator

Supplementary Table S2. Perinatal outcomes in births to asymptomatic women by dominant variant.

Table S2. Perinatal outcomes in births to women with asymptomatic SARS-CoV-2 admitted to hospital by dominant variant, from March 1, 2020, to March 31, 2022, United Kingdom.

SARS-CoV-2 dominant variant	Wild-type (N=1357)	Alpha (N=2125)	Delta (N=1781)	Omicron (N=3972)
	n (%)	n (%)	n (%)	n (%)
Stillbirth -no. (%)	6 (0.4)	22 (1.0)	27 (1.5)	24 (0.6)
Preterm births* -no. (%)				
<34 weeks	44 (3.3)	91 (4.3)	107 (6.1)	148 (3.8)
34^{+0} –36^{+6} weeks	97 (7.2)	178 (8.4)	131 (7.4)	365 (9.3)
Admission to Neonatal Unit† - no. (%)	128 (9.5)	245 (11.7)	203 (11.6)	388 (9.8)
Neonatal Death‡ - no. (%)	2 (0.1)	7 (0.3)	8 (0.5)	14 (0.4)

* 64 infants born to asymptomatic women had missing data for gestational age at birth

† 74 infants born to asymptomatic women had missing data for admission to neonatal unit.

‡ 59 infants born to asymptomatic women had missing data for neonatal death

Supplementary Table S3. Crude risk ratios for perinatal outcomes in births to symptomatic women by dominant variant.

Table S3. Crude risk ratios for perinatal outcomes to women admitted to hospital with symptomatic SARS-CoV-2 by dominant variant, March 1, 2020, to March 31, 2022, United Kingdom

SARS-CoV-2 dominant variant	n (%)	Crude RR (95%CI)
Stillbirth -no (%)		
Wild-type	15 (1.0)	[Ref]
Alpha	21 (1.1)	1.06 (0.55- 2.05)
Delta	62 (2.5)	2.39 (1.36- 4.21)
Omicron	13 (1.0)	0.95 (0.44- 2.04)
Preterm birth* -no (%)		
<34 weeks		
Wild-type	113 (7.9)	[Ref]
Alpha	177 (9.4)	1.21 (0.94- 1.55)
Delta	269 (10.9)	1.49 (1.18- 1.87)
Omicron	58 (4.5)	0.53 (0.38- 0.74)
34 –36 weeks		
Wild-type	169 (11.8)	[Ref]
Alpha	225 (11.9)	1.03 (0.83- 1.27)
Delta	354 (14.4)	1.31 (1.07- 1.59)
Omicron	142 (10.9)	0.87 (0.69- 1.11)
Neonatal Unit admission† -no (%)		
Wild-type	264 (18.6)	[Ref]
Alpha	391 (20.9)	1.12 (0.98- 1.29)
Delta	529 (21.9)	1.18 (1.03- 1.34)

Omicron	155 (12.0)	0.64 (0.54- 0.77)

Abbreviations: risk ratio (RR), confidence interval (CI)

[*] 45 infants born to symptomatic women had missing data for gestational age at birth

[†] 112 infants born to symptomatic women had missing data for admission to neonatal unit.

[‡] 77 infants born to symptomatic women had missing data for neonatal death

Supplementary Table S4. Crude risk ratios for perinatal outcomes in births to symptomatic women by severity of maternal infection.

Table S4. Crude risk ratios for perinatal outcomes in births to women with symptomatic SARS-CoV-2 by severity of maternal infection, March 1, 2020, to March 31, 2022, United Kingdom.

Severity	Mild infection (N=4805) n (%)	Moderate to severe infection (N=2311) n (%)	RR (95% CI)
Stillbirth	71 (1.5)	40 (1.7)	1.18 (0.80 to 1.74)
Preterm birth*			
<34 weeks	245 (5.2)	372 (16.4)	4.10 (3.45 to 4.87)
34 to 36^6 weeks	478 (10.1)	412 (18.1)	2.33 (2.02 to 2.69)
Neonatal unit admission†	589 (12.4)	750 (33.0)	2.65 (2.41 to 2.92)

Abbreviations: risk ratio (RR), confidence interval (CI)

Moderate to severe maternal infect1.ion was defined according to modified WHO criteria as maternal death, maternal intensive care admission, peripheral oxygen saturation below 95% at admission, pneumonia on radiological imaging or respiratory support (either oxygen supplementation, non-invasive ventilation (high flow nasal oxygen or continuous positive airway pressure), mechanical ventilation or extracorporeal membrane oxygenation (ECMO).

* 45 infants born to symptomatic women had missing data for gestational age at birth

† 112 infants born to symptomatic women had missing data for admission to neonatal unit.

Supplementary Table S5. Perinatal outcomes in births to asymptomatic women by maternal vaccine status.

Table S5. Perinatal outcomes in births to asymptomatic women admitted to hospital with SARS-CoV-2 by maternal vaccine status, from January 1, 2021, to March 31, 2022, United Kingdom.

Vaccination status	Unvaccinated (N=4070)	Status unknown (N=1639)	1 dose (N=622)	2 doses (N=826)	3 doses (N= 135)
Stillbirth - no. (%)	32 (0.8)	17 (1.0)	9 (1.5)	7 (0.9)	0
Preterm births* - no. (%)					
<34 weeks	189 (4.7)	60 (3.7)	31 (5.0)	35 (4.3)	4 (3.0)
34^{+0} –36^{+6} weeks	331 (8.2)	131 (8.1)	62 (10.0)	80 (9.7)	12 (8.9)
Admission to Neonatal Unit† - no. (%)	442 (10.9)	160 (9.9)	59 (9.6)	93 (11.4)	12 (9.0)
Neonatal Death‡ - no. (%)	17 (0.4)	6 (0.4)	1 (0.2)	2 (0.2)	1 (0.7)

*55 infants born to asymptomatic women had missing data for gestational age at birth

† 62 infants born to asymptomatic women had missing data for admission to neonatal unit.

‡ 49 infants born to asymptomatic women had missing data for neonatal death

References

1. Vousden N, Ramakrishnan R, Bunch K, Quigley M, Kurinczuk J, Knight M. Severity of maternal infection and perinatal outcomes during periods in which Wildtype, Alpha and Delta SARS-CoV-2 variants were dominant: Data from the UK Obstetric Surveillance System national cohort. BMJ Medicine 2022;1(1).
2. Knight M, Bunch K, Vousden N, et al. Characteristics and outcomes of pregnant women admitted to hospital with confirmed SARS-CoV-2 infection in UK: national population based cohort study. BMJ 2020;369:m2107. DOI: 10.1136/bmj.m2107.
